# Supplementary material for: Impact of COVID-19 and Antibiotic Treatments on Gut Microbiome: A Role for Enterococcus spp
Source: Biomedicines. 2022 Nov 2;10(11):2786. doi: 10.3390/biomedicines10112786 (PMC9687172; doi:10.3390/biomedicines10112786)
Supplement: Supplementary file 1 [file biomedicines-10-02786-s001.zip › biomedicines-1962242-supplementary.pdf]

Supplementary Materials

Table S1. Concomitant infections in COVID-19 patients. Only microbiologically documented (e.g., fungal or bacterial bloodstream infections, bacterial pneumonia) infections are reported.

Pathogen	N
Pseudomonas aeruginosa	1
Streptococcus pneumoniae	5
Acinetobacter baumannii	1
Staphylococcus epidermidis	1
Escherichia coli	1
Enterobacter aerogenes	1
Staphylococcus aureus	1
Stenotrophomonas maltophilia	1
Streptococcus agalactiae	1
Candida albicans	2

Table S2. Type of treatments and treatment combinations

Treatment	N (%)
**Any type (n=82)**	
- Nor AT neither NACT	15 (18)
- AT only	9 (11)
- AT + NACT	40 (49)
- NACT only	18 (22)
**COVID-19 related treatment**	
- Hydroxychloroquine (HCQ)	27 (33)
- Lopinavir/ritonavir (LPV/r)	22 (27)
- Corticosteroids	45 (55)
- Remdesivir	12 (14)
- Tocilizumab	16 (20)
- Heparin	21 (26)
**Antibiotic treatment (AT)**	
- Any	49 (60)
- Combination of 2 or more	11 (13)
- Beta-lactams	34 (41)
- Anti-intracellular	25 (30)
- Anti-Gram-positive	6 (7)
**Combinations of AT + NACT**	
- AT + hydroxychloroquine	14 (17)
- AT + heparin	5 (6)
- AT + LPV/r	11 (13)
- AT + remdesivir	3 (3)
- AT + corticosteroids	22 (27)
- AT + tocilizumab	6 (7)
- HCQ + LPV/r	21 (26)
- HCQ + tocilizumab	10 (12)
- HCQ + corticosteroids	16 (19)
- Heparin + remdesivir	9 (11)
- Heparin + corticosteroids	19 (23)

-	Remdesivir + corticosteroids	12 (14)
-	LPV + corticosteroids	15 (18)
-	LPV + tocilizumab	8 (10)

Treatment or treatment combinations were considered if administered to 3 or more patients. Each treatment or treatment couple is considered separately therefore the total number exceeds 82 patients.

Table S3. Logistic regression model used to investigate the association between antibiotic use and clinical variables. Clinical and laboratory parameters were compared between patients receiving therapeutic regimens including antibiotics (N=49) and those not receiving antibiotic-based treatments (N=15)

Parameter	OR	95% CI	P value
Age (years)	1.04	0.99 - 1.08	1.08
Gender	0.51	0.12 - 2.13	0.36
Length of stay (days)	0.95	0.89 - 1.02	0.12
O2 support	1.08	0.27 - 4.34	0.92
D-dimer (ng/ml)	1.00	0.99 - 1.00	0.10
Ferritin (ng/ml)	1.00	0.99 - 1.00	0.62
IL6 (pg/ml)	1.00	0.89 - 1.01	0.16

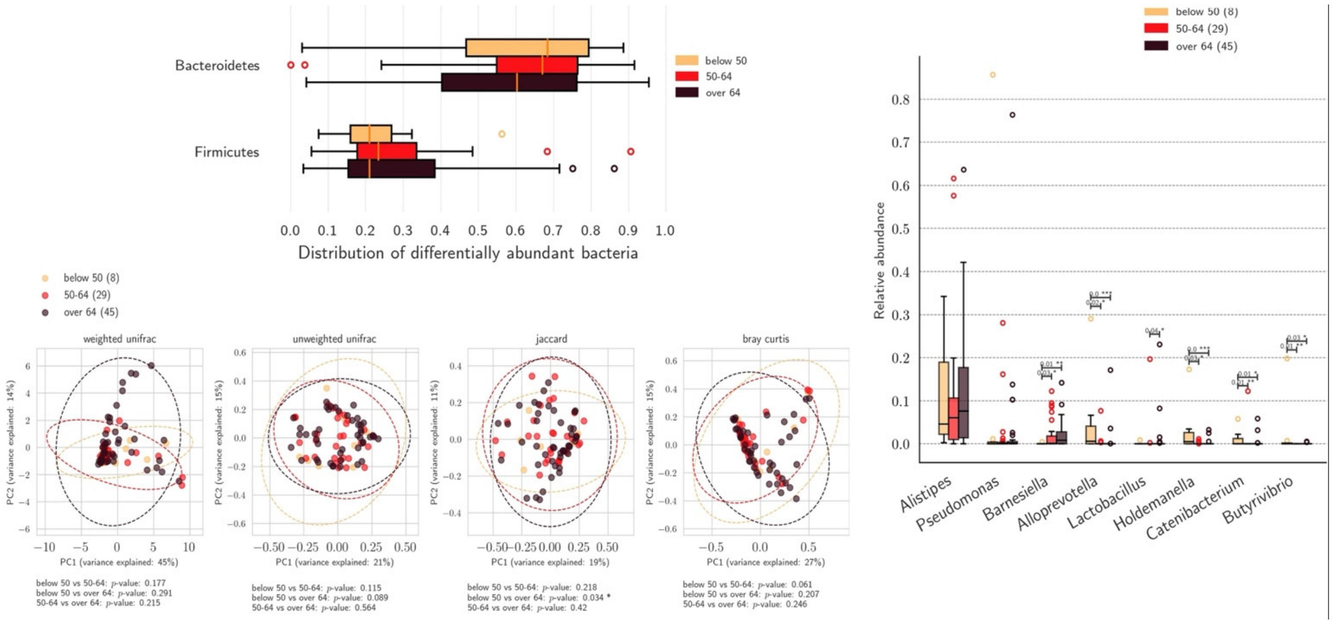

Figure S1. Age groups. No differences were shown for overall gut microbiome composition at a phylum level, while there was a significant difference in Jaccard beta-diversity in patients aged < 50 and > 64 years. Pathogen relative abundance at a genus level showed no clear directions according to opportunistic pathogens or symbionts according to the age group. Statistical significance reported as *p ≤ 0.05, **p ≤ 0.01, ***p ≤ 0.001

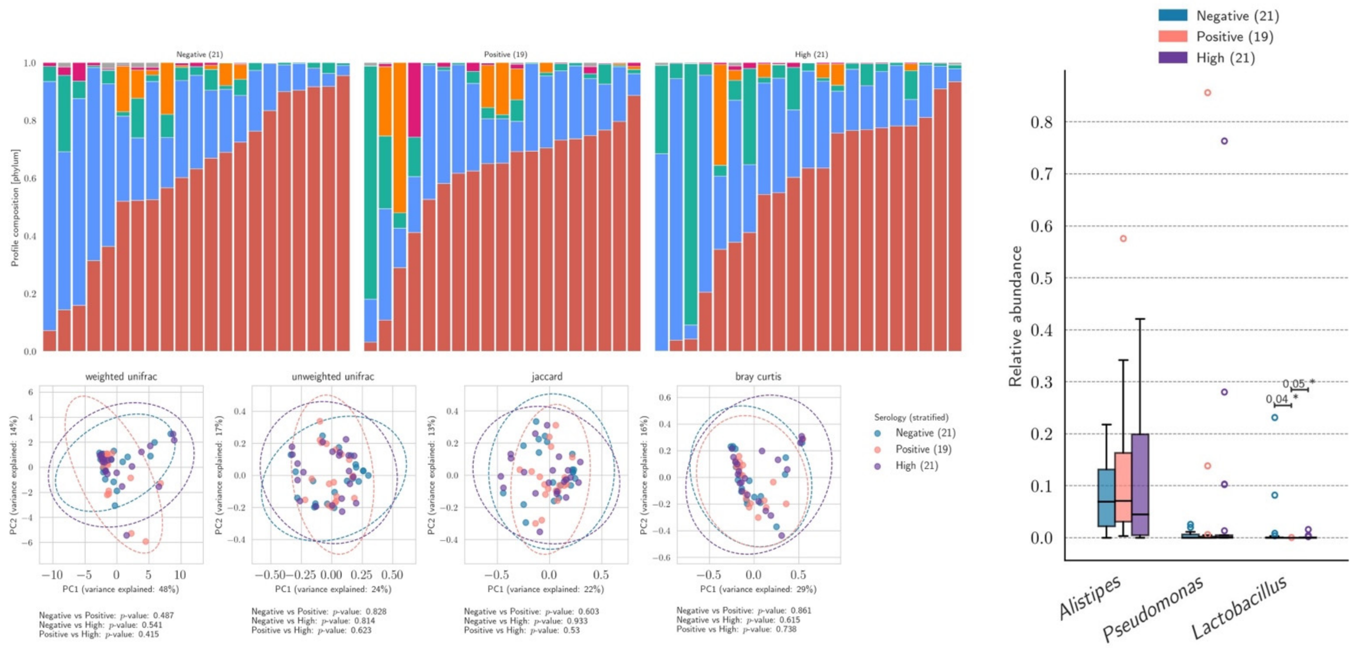

Figure S2. Antibody response. No differences were shown for overall gut microbiome composition at a phylum level and beta-diversity based on the antibody specific response to SARS-CoV-2. Pathogen relative abundance at a genus level showed lower Lactobacillus relative abundance in patients with moderate serological response compared to the other groups. Statistical significance reported as *p ≤ 0.05.

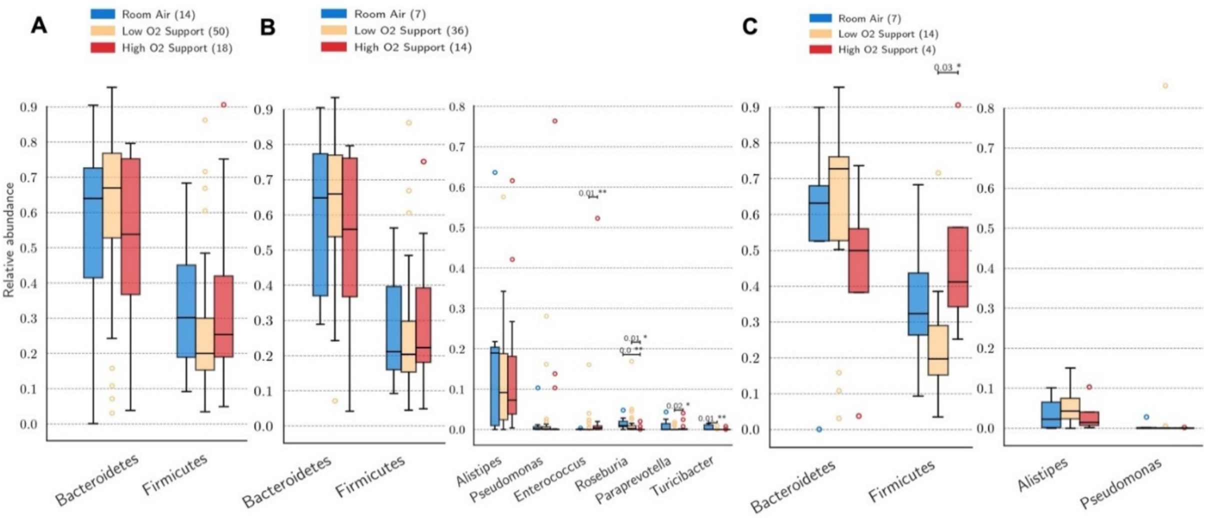

Figure S3. O_2 therapy. A. Gut microbiome relative abundance at the phylum level according to the levels of O_2 therapy did not show significant differences in the distribution of Bacteroidetes and Firmicutes. **B.** Differential abundance at phylum and genus level in males **C.** Differential abundance at phylum and genus level in females. Statistical significance reported as *p ≤ 0.05, **p ≤ 0.01

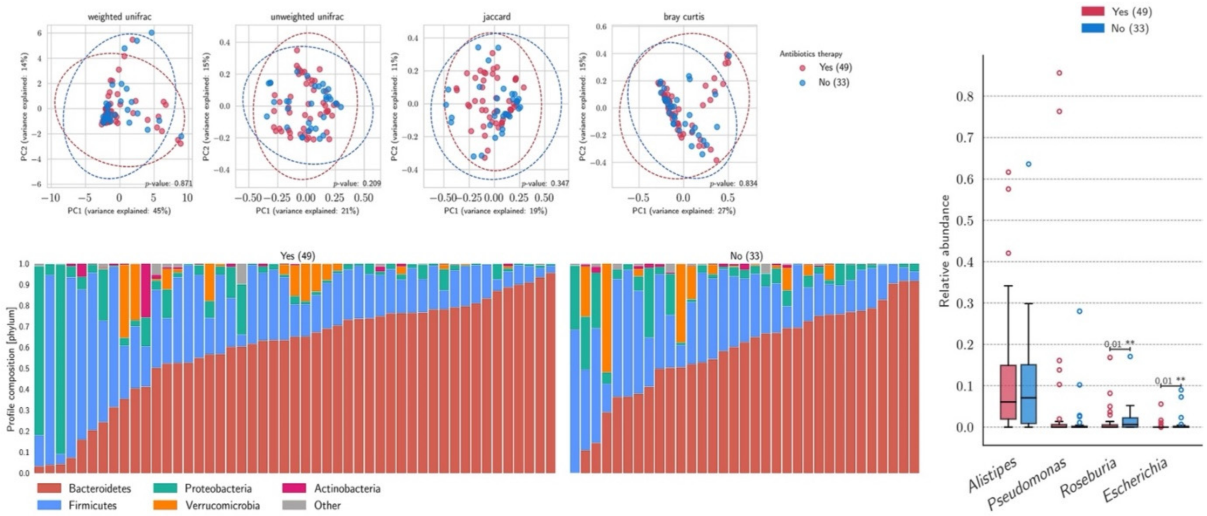

Figure S4. **Antibiotic treatment**. No differences were shown for overall gut microbiome composition at a phylum level and beta-diversity according to antibiotic treatment. Pathogen relative abundance at a genus level showed higher Roseburia and Escherichia abundance in patients not receiving antibiotic therapy compared to those receiving antibiotics during hospitalization for COVID-19. Statistical significance reported as **p ≤ 0.01

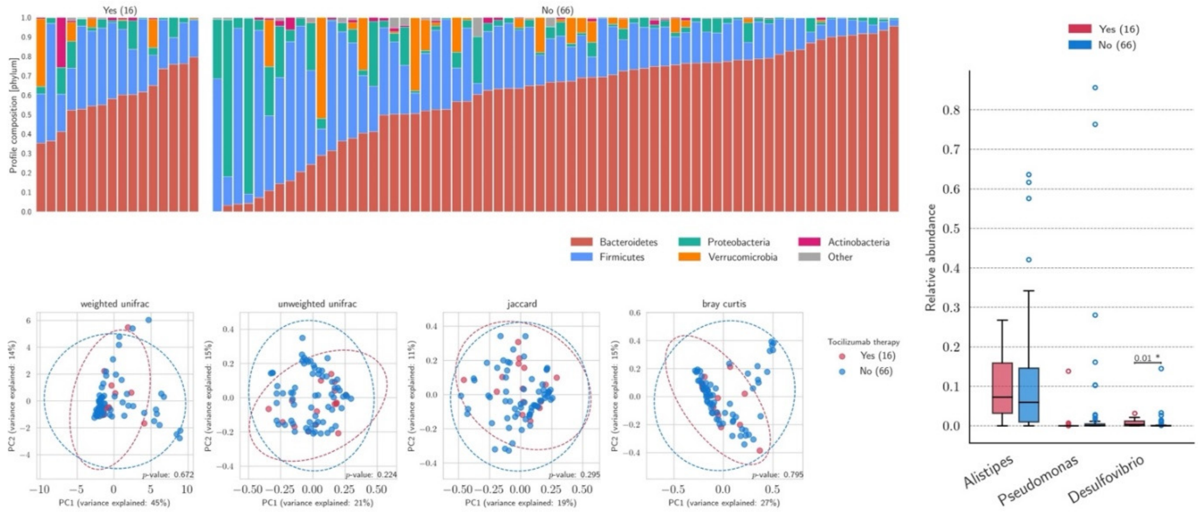

Figure S5. **Tocilizumab.** No differences were shown for overall gut microbiome composition at a phylum level and beta-diversity based on tocilizumab treatment. Pathogen relative abundance at a genus level showed increased *Desulfovibrio* relative abundance in patients receiving tocilizumab compared with those who did not receive it. Statistical significance reported as *p ≤ 0.05
